# A Case of Clear Cell Papillary Renal Cell Tumor: A Rare Entity With an Unusual Presentation

**DOI:** 10.1002/ccr3.72810

**Published:** 2026-05-26

**Authors:** Mehdi Montazer, Naser Tayyebi Meibodi, Salman Soltani, Sarvin Beigi

**Affiliations:** ^1^ Department of Pathology Mashhad University of Medical Sciences Mashhad Iran; ^2^ Department of Urology Mashhad University of Medical Sciences Mashhad Iran

**Keywords:** chronic kidney failure, cystic kidney disease, immunohistochemistry, renal cell carcinoma

## Abstract

Clear cell papillary renal cell Tumor (CCPRCT) is a rare variant of renal cell carcinoma that typically arises in a nonfunctional kidney. We report a case of CCPRCT in a 49‐year‐old Iranian male who presented with a mural nodule within a large cystic mass in his nonfunctional left kidney, and we discuss the relevant diagnostic challenges. Immunohistochemistry revealed diffuse and intense positivity for CK7 and carbonic anhydrase IX in a cuplike distribution, p53 positivity, and no expression of AMACR or CD10. Ki67 staining showed approximately 5% positivity in neoplastic cells. The differential diagnoses include clear cell and papillary renal cell carcinomas and papillary adenoma, all of which share overlapping clinical, imaging, histopathologic, and immunophenotypic features with CCPRCT. Accurate histological and immunophenotypic evaluation is crucial, as CCPRCT is clinically indolent, and correct diagnosis significantly influences patient outcomes and quality of life.

## Key Clinical Message

Clear cell papillary renal cell tumor can present as a subtle mural nodule within a large renal cyst, creating significant diagnostic uncertainty. This case highlights the importance of meticulous imaging review and maintaining a broad differential for cystic renal lesions, particularly in patients with complex renal histories such as transplantation.

## 1 Introduction

Clear cell papillary renal cell carcinoma (CCPRCT) was recently added to the World Health Organization classification of renal tumors as an entity with exceptionally indolent behavior, representing the fourth most common type of renal cell carcinoma. While the immunohistopathological and genetic profiles are well established, the clinical and long‐term behavior is still insufficiently understood [1, 2, 3].

CCPRCT presents as a solid or cystic mass and is morphologically characterized by the presence of papillary or tubular structures lined by a single layer of clear cells with low‐grade nuclear features [4, 5]. The neoplastic cells differ from those of clear cell renal cell carcinoma (CCRCC) and papillary renal cell carcinoma (PRCC) due to their distinct immunophenotype, which includes diffuse and intense CK7 positivity, carbonic anhydrase IX positivity with a cuplike quality, P53 overexpression, constant negativity for AMACR, and absent or focal positivity for CD10 [1, 2, 6, 7].

Herein, we report a case of CCPRCT presenting as a mural nodule within a large cystic mass and discuss the relevant diagnostic challenges.

## 2 Case History/Examination

The patient was a 49‐year‐old Iranian male living in Mashhad who was referred to Imam‐Reza Hospital, Mashhad University of Medical Sciences, in November 2020 with a two‐month history of recent left flank pain, nausea, and vomiting. His past medical history was remarkable for right kidney transplantation 15 years prior and long‐standing hypertension.

## 3 Differential Diagnosis, Investigations and Treatment

**FIGURE 1:**
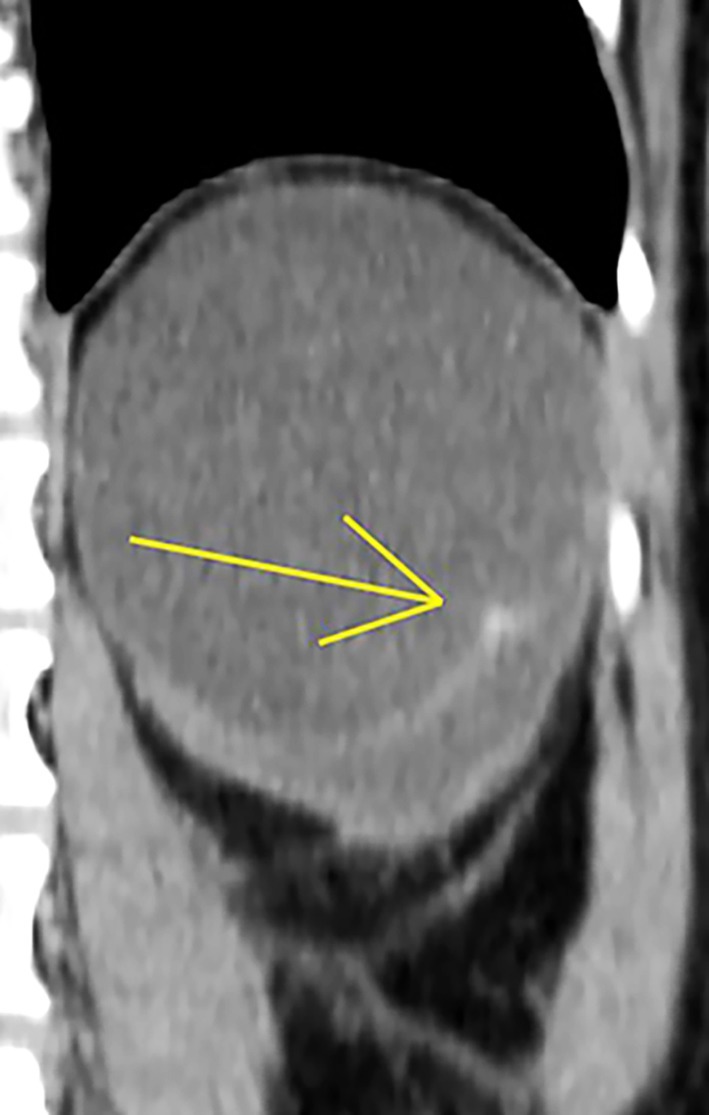
Computed tomography scan showing a cystic lesion with a contrast‐enhanced mural nodule within the left kidney.

Abdominal ultrasonography and computed tomography scans revealed a double‐cavity cystic structure measuring approximately 121 × 98 mm within the left non‐functioning kidney (Figure 1). A small contrast‐enhanced mural nodule was also identified on retrograde examination of the images, which had originally been overlooked. A differential diagnosis of benign cystic kidney disease versus cystic renal cell carcinoma was considered. The patient subsequently underwent a left radical nephrectomy.

## 4 Conclusion and Results

**FIGURE 2:**
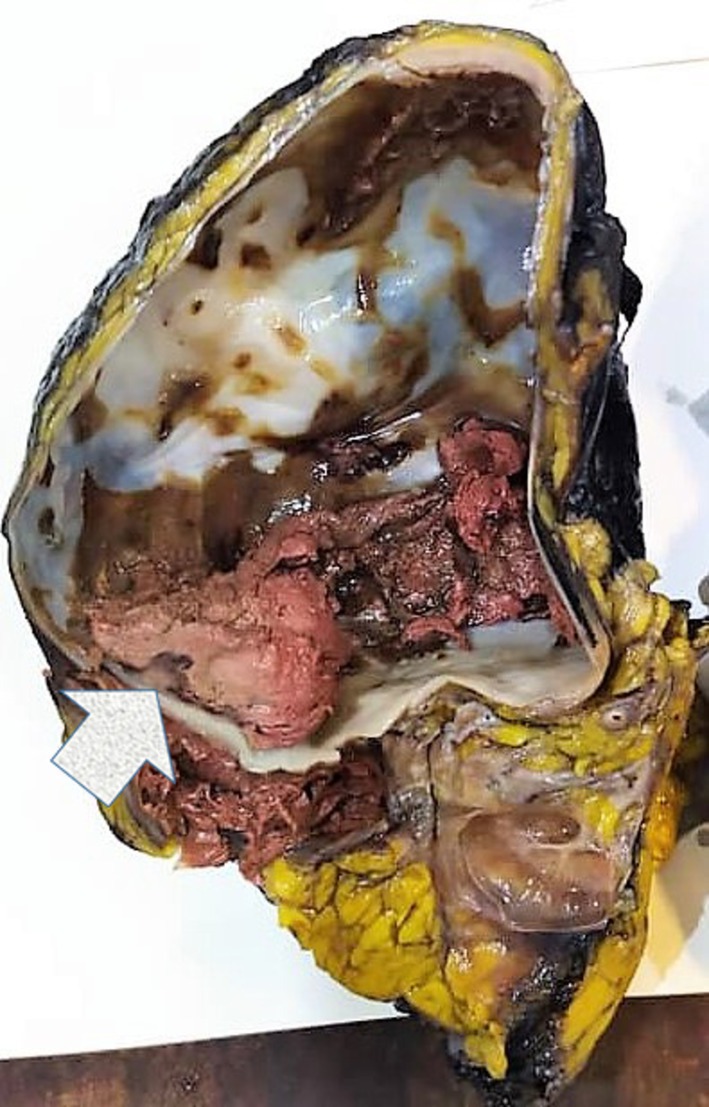
Gross examination of the kidney. Arrow points to the mural nodule with its adjacent blood clot formation.

On gross examination, the kidney and the adjacent fat weighed 995 g and measured 18 × 11.5 × 10.5 cm. Cut sections showed a thick‐walled double‐cavity cystic lesion located in the upper and middle parts of the kidney, measuring 15 × 11 cm. A mural nodule measuring 1 × 0.5 × 0.5 cm, surrounded by a blood clot, was noted (Figure 2). The lower pole of the kidney was unremarkable.

**FIGURE 3:**
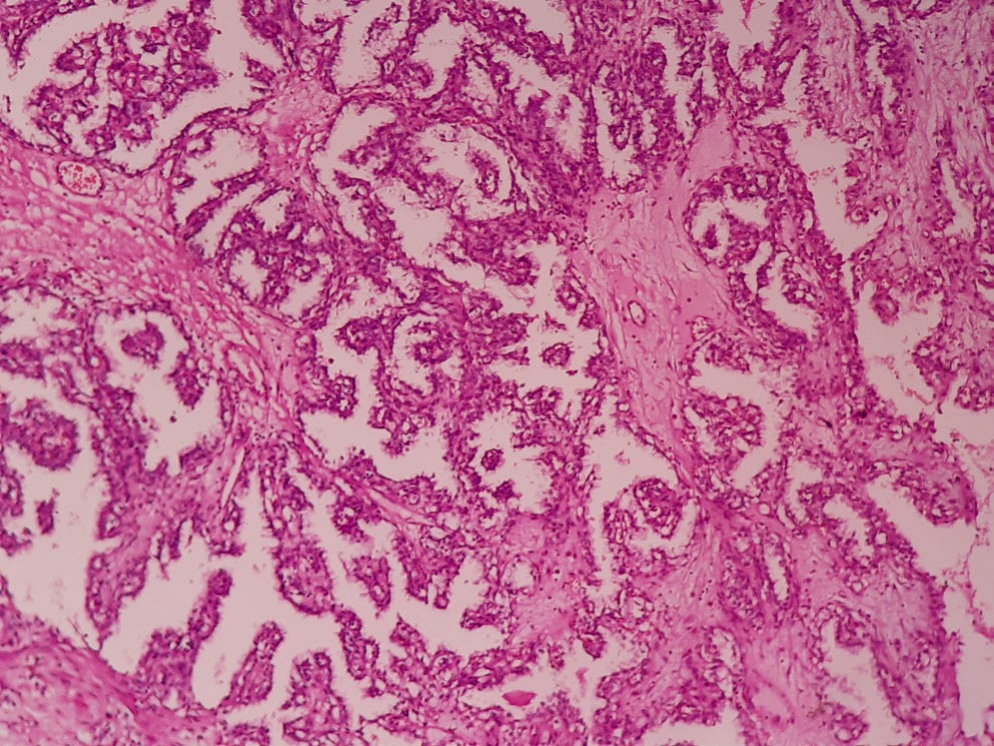
Microscopic examination of the lesion. Papillary formations lined by clear cell epithelium are present (H&E stain, original magnification: ×100).

Microscopically, sections showed a cystic lesion with focal epithelial lining containing necrotic material and blood clot. The mural nodule exhibited a tubulo‐papillary architecture lined by cuboidal to low columnar cells with low‐grade nuclear features, including small nucleoli (Figure 3). Focally, the nuclei were arranged in the middle of the cells, away from the basement membrane. No foamy macrophages were noted. The uninvolved renal parenchyma revealed glomerulosclerosis, tubular atrophy, interstitial fibrosis, chronic inflammation, and a papillary adenoma.

**FIGURE 4:**
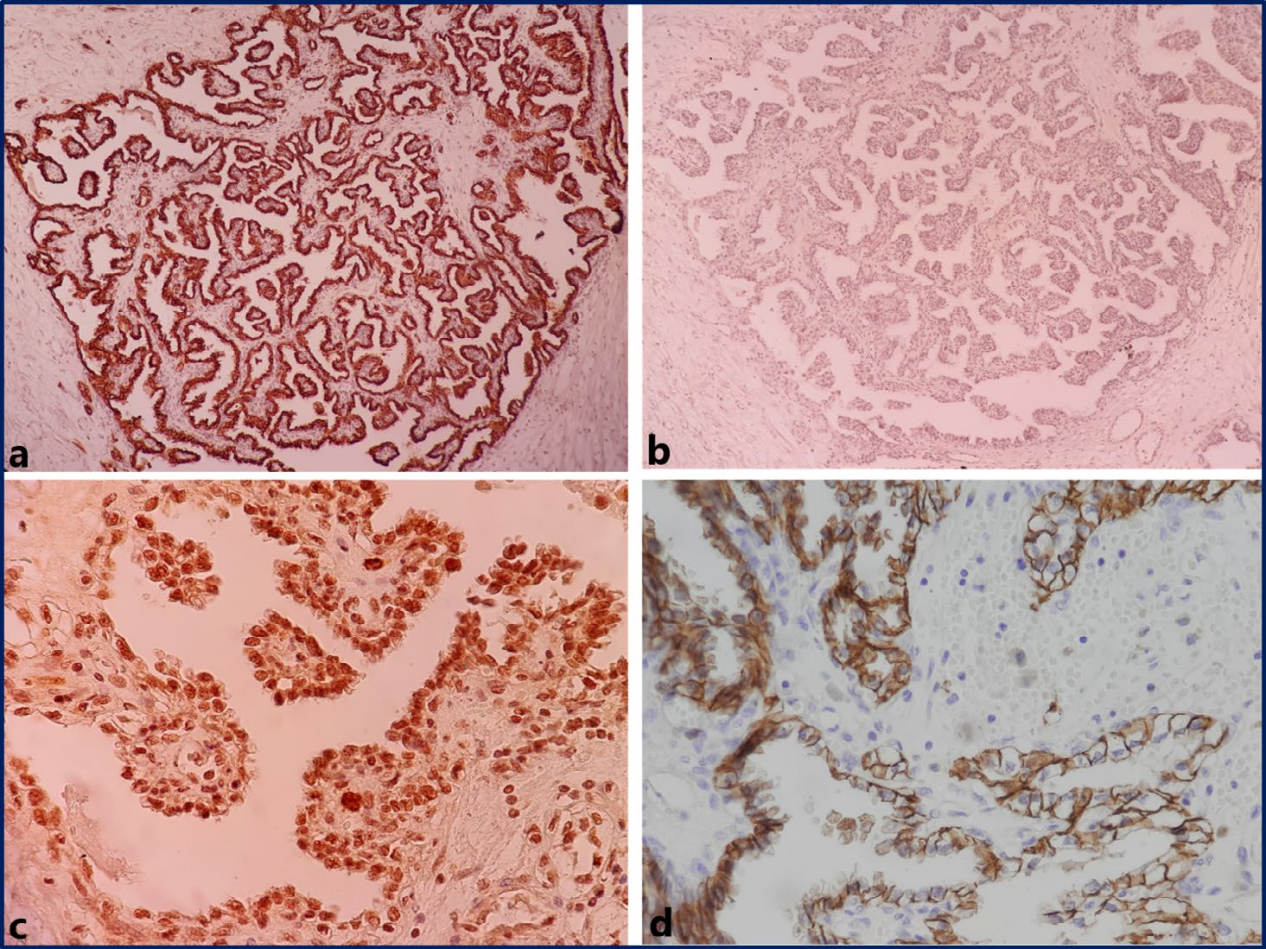
Immunohistochemistry results. Diffuse and strong staining for CK7 (a), negative AMACR (b), and high expression of p53 (c), carbonic anhydrase IX (cuplike pattern) (d) (original magnifications: ×100 for (a) and (b), ×400 for (d) and (c)).

Immunohistochemically, the specimen diffusely and strongly expressed CK7, P53, and carbonic anhydrase IX in a cuplike pattern, whereas CD10 and AMACR were non‐reactive (Figure 4). The MIB1 proliferation index was approximately 5%. A final diagnosis of CCPRCC was made.

## 5 Discussion

Clear cell papillary renal cell carcinoma is a variant of renal cell carcinoma, first described by Tickoo et al. in end‐stage renal disease (ESRD) patients in 2006. However, CCPRCC is now acknowledged to occur in healthy functional kidneys as well [4, 5].

CCPRCC shares certain clinical, imaging, histopathologic, and immunophenotypic features with other more prevalent renal tumors, such as clear cell, papillary, and cystic RCCs, which renders it a potential diagnostic challenge. It is noteworthy that a proper distinction is crucially important because most mimickers harbor a worse prognosis than CCPRCT. In fact, CCPRCT is generally known as a low‐grade indolent neoplasm, and there have been no recorded cases of metastasis to date [8, 9].

In our patient, the postoperative course was uneventful, and follow‐up imaging demonstrated no evidence of recurrence or metastasis, consistent with the expected benign behavior of CCPRCC.

Radiologically, CCPRCTs are indistinguishable from other renal cell carcinomas. They usually appear as solid contrast‐enhanced heterogeneous masses; however, exceptionally rare cases, such as ours, have presented as a mural nodule within an otherwise cystic structure [1, 2, 8].

Histomorphologically, CCPRCT exhibits features intermediate between clear cell renal cell carcinoma (CCRCC) and papillary renal cell carcinoma (PRCC), characterized by low nuclear grade, a tubulo‐papillary arrangement of clear epithelial cells, and predominantly linear nuclear alignment away from the basement membrane. Necrosis, lymphovascular invasion, and extension into the renal sinus or extra‐renal tissue are absent [1, 6]. Given these overlapping histologic characteristics, immunohistochemistry plays an essential role in differentiating these entities. CCPRCTs are typically CK7‐positive and CD10‐negative, which distinguishes them from CK7‐negative CD10‐positive CCRCTs. Furthermore, in contrast to PRCCs, CCPRCTs are non‐reactive to AMACR [1, 4].

Multilocular cystic renal neoplasm of low malignant potential (MCRNLMP) can mimic clear cell papillary renal cell tumor (CCPRCT). These entities are distinguished by their histologic features and immunoprofiles: MCRNLMP typically shows CD10 positivity, whereas CCPRCT is characteristically CK7‐positive with carbonic anhydrase IX staining in a cuplike pattern [10].

Another possible differential diagnosis is renal papillary adenoma. By definition, renal papillary adenomas are small (< 15 mm), unencapsulated bland tubulo‐papillary structures usually found incidentally within the renal parenchyma. They have been proposed as precursors for papillary renal cell carcinomas; thus, both papillary adenomas and PRCCs display immunohistochemical expression for AMACR. However, like CCPRCTs, papillary adenomas arising in a background of acquired polycystic kidney disease may not express AMACR, which presents a diagnostic pitfall if other gross and microscopic findings are ignored [11].

In summary, clear cell papillary renal cell carcinomas are rare renal tumors that may unusually present as a small mural nodule within a large cyst, which can easily be missed in radiologic or gross examinations. Since CCPRCTs behave relatively benignly in their clinical course, their more malignant mimickers must be accurately ruled out, which is best achieved through an appropriate immunohistochemistry panel.

## Author Contributions

**Mehdi Montazer:** data curation, formal analysis, methodology, software, supervision, validation, visualization. **Naser Tayyebi Meibodi:** methodology, supervision, validation. **Salman Soltani:** data curation, resources. **Sarvin Beigi:** data curation, investigation, project administration, software, writing – original draft, writing – review and editing.

## Funding

The authors have nothing to report.

## Consent

The authors declare that written informed consent was obtained for the publication of this manuscript and accompanying images using the consent form provided by the Journal.

## Conflicts of Interest

The authors declare no conflicts of interest.

## Data Availability

The data supporting the findings of this case report are wrapped entirely within the article text and are available from the corresponding author upon reasonable request.
